# Tuberculosis among migrant workers in Taiwan

**DOI:** 10.1186/s12992-019-0461-2

**Published:** 2019-02-28

**Authors:** Chia-Wen Lu, Yi-Hsuan Lee, Yu-Hao Pan, Hao-Hsiang Chang, Yi-Chun Wu, Wang-Huei Sheng, Kuo-Chin Huang

**Affiliations:** 1 Department of Family Medicine, National Taiwan University Hospital, Taipei, Taiwan; 2 Department of Family Medicine, College of Medicine, National Taiwan University, Taipei, Taiwan; 3 Department of Family Medicine, National Taiwan University Hospital Bei-Hu Branch, Taipei, Taiwan; 4 Centers for Disease Control, Taipei, Taiwan; 5 Department of Medical Education, National Taiwan University Hospital, Taipei, Taiwan

**Keywords:** Tuberculosis, Migrant workers, Latent tuberculosis infection, Foreigner workers

## Abstract

**Background:**

Although the worldwide incidence of tuberculosis (TB) has been slowly decreasing, the migrant workers remains an important gap for regional TB control. In Taiwan, the numbers of the migrant workers from countries with high TB incidence increase significantly in past decades and the impact on public health remains unknown. This study aimed to explore the difference of TB incidence between Taiwanese and the migrant workers.

**Methods:**

The migrant workers are obligated to receive pre-arrival, post-arrival and regular chest X-ray screening during their stay in Taiwan. We retrospectively collected these data extracted from the Alien Workers Health Database in Centers for Disease Control, Taiwan from Jan. 1, 2004 to Dec. 31, 2013. Poisson regression models were used to compare the hazard ratios of TB between Taiwanese and the migrant workers after adjusting gender and age groups.

**Results:**

The total migrant workers in Taiwan reached 314,034 persons in 2004 and 489,134 persons in 2013, accounting for 2% of Taiwan population. The TB incidence of migrant workers was similar to Taiwanese (53–73.7 per 10^5^ vs 45.5–76.8 per 10^5^). Comparing with Taiwanese, the TB risk was significantly lower in male migrant workers (HR: 0.76; 95% CI: 0.70–0.83, *P* < 0.001), but higher in female migrant workers (HR: 1.40; 95% CI: 1.35–1.46, *P* < 0.001). Besides, we found that the TB risk in migrant workers was 5.30-fold (95% CI: 4.83–5.83, *P* < 0.001) in youngest group (≤24 year-old) comparing with Taiwanese.

**Conclusions:**

Migrant workers in Taiwan have higher TB incidence than Taiwanese in young groups, especially in females. The mainstay young laborers with latent tuberculosis infection risk is an important vulnerability for public health. Further investigation and health screening are warranted.

## Background

Tuberculosis (TB) is the ninth leading cause of death worldwide, with most of them could be prevented with early diagnosis and appropriate treatment [1]. In 2016, there were 6.3 million new cases of TB globally [1]. Among the new cases, the top two incidence countries were India and Indonesia and the most TB-resistant countries were India and China [1]. Similarly, TB is one of the top infectious diseases and an important public health concern in Taiwan. The Bureau of National Health Insurance (NHI) in Taiwan introduced the no-notification-no-reimbursement policy to enhance TB notification and to decrease the incidence of TB since 1997 [2]. Accordingly, the NHI, which enrolled up to 99% of Taiwanese, has reached the TB notification rate more than 97% since 2007 [3, 4]. Also, the TB numbers (incidence) in Taiwan dropped from more than 16,000 cases (72 cases per 10^5^ persons) in 2005 to less than 10,000 cases (41 cases per 10^5^ persons) in 2017 [5].

Previous studies have shown that migrant workers from countries with a high incidence of TB have a significant impact on the epidemic of TB in low-incidence countries [6–8]. Pre-entry screening programs for tuberculosis in migrant workers is believed to be a high yield policy for active TB [9], though the risk of latent TB reactivation is persistent [10, 11]. The average annual TB notification rate is reported to be higher in the foreign-born population than the Taiwan-born population [12]. In addition, the numbers of the migrant workers in Taiwan from countries with high TB incidence increase significantly in past decades [5]. Although the migrant workers have to receive pre-entry screening and post-arrival regular health check-up, to date, little is known about the TB incidence and the characteristics of TB population of the migrant workers in Taiwan. Therefore, this study aimed to explore the difference of TB incidence between Taiwanese and migrant workers. Also, the study aimed to compare the gender and age differences of TB burden in Taiwanese with the migrant workers.

## Methods

### Design

The profile of the general population of Taiwanese was obtained from the official publications of the Ministry of the Interior, Taiwan [13]. The incidence rate of TB detected in each origin countries were estimated by the WHO [14]. The profile of migrant workers was extracted from the National Immigration Agency where the number of persons entering and leaving Taiwan were recorded and stratified by country of origin, gender and age [15]. Since TB is a notifiable disease by the Law on the Control of Communicable Diseases in Taiwan, data of all TB cases in Taiwanese notified between January 1, 2004 and December 31, 2013 were obtained from the National TB Registry [16]. All migrant workers were obligated to receive pre-arrival, post-arrival (within 3 days after arrival) and regular chest X-ray screening (6th, 18th and 30th month after arrival) during their stay in Taiwan [17]. Besides, all migrant workers were seronegative of human immunodeficiency virus (HIV) before their entry and were excluded from the study once diagnosis of HIV infection. Also, we retrospectively collected TB cases from Alien Workers Health Database in Centers for Disease Control, Taiwan [18]. The epidemiological records in their native-born countries were extracted from annual reports in World Health Organization [19]. The design of the study was approved by the Research Ethics Committee in National Taiwan University Hospital (201807018W) before the study was conducted.

### Case definition

TB in Taiwan was diagnosed by two approaches: 1) laboratory diagnosis: human specimens which was smear-positive for acid-fast bacilli and/or culture-positive for *Mycobacterium tuberculosis* including sputum, body fluid and tissue; 2) clinical diagnosis by specialists: the clinical and radiographic manifestations were compatible with TB, the patient was received detailed evaluation to exclude other diagnoses, and the patient has initiated treatment with more than one anti-tuberculosis drugs [19].

### Statistical analysis

The baseline categorical data were presented by frequency with percentages. The TB incidence was defined as the number of events divided by the follow-up person-years, which were calculated as the time from 2004 to the diagnosis of TB, death or the end of 2013. We used Poisson regression models to assess hazard ratios (HRs) and 95% confidence intervals (CIs) of TB for the Taiwanese compared to the migrant workers after adjusting gender and age groups respectively. All 2-sided *p* values were presented. All analyses were computed using the SAS version 9.4 (SAS Institute Inc. Cary, North Carolina).

## Results

**Fig. 1:**
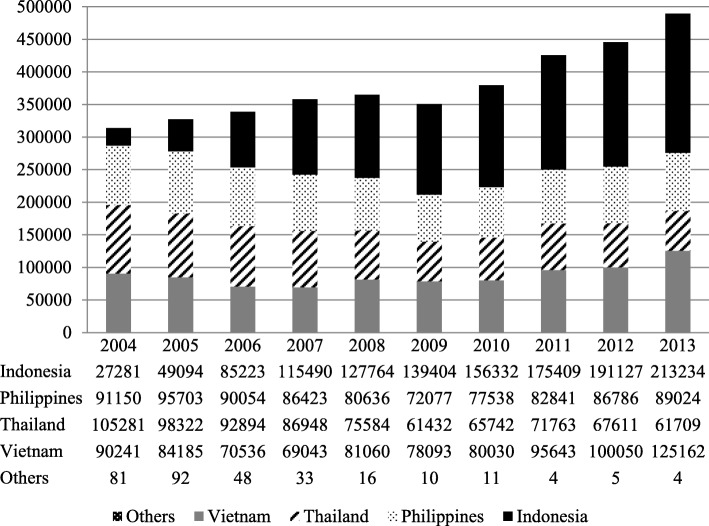
Annual numbers of the migrant workers in Taiwan, 2004–2013

**Fig. 2:**
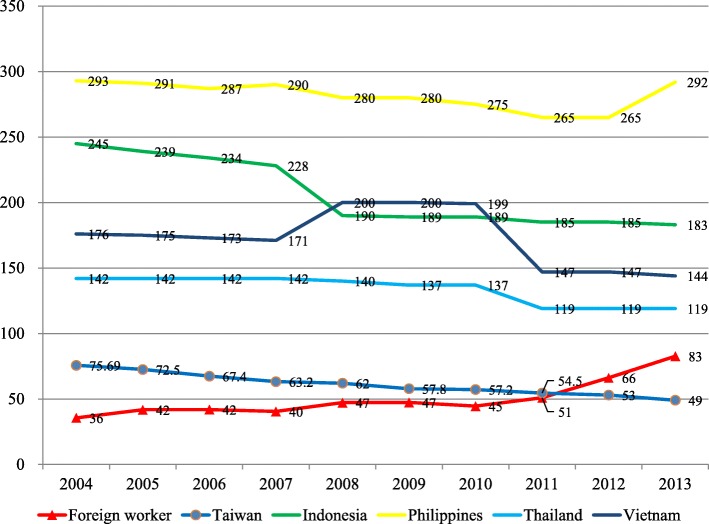
Tuberculosis incidence (persons per 105) among the migrant workers, their native countries and Taiwan

The total migrant workers in Taiwan reached 314,034 persons in 2004 and 489,134 persons in 2013, accounted for 2% of Taiwan population. The mainly import countries were Indonesia, Philippines, Thailand, and Vietnam in Fig. 1. From 2004 through 2013, 2256 new cases of TB were found among migrant workers during their regular health examination in Tables 1 and 2. The TB incidence of the migrant workers was similar to Taiwanese. (53–73.7 per 10^5^ vs 45.5–76.8 per 10^5^) From 2004 to 2013, the annual TB incidence in Taiwanese has been decreasing whereas the annual TB incidence in migrant workers has been increasing in Fig. 2. Therefore, the TB incidence of migrant workers exceeded Taiwanese since 2012.

**Table 1:** The incidence of tuberculosis among Taiwanese and the foreign laborers during 2004–2013

	Taiwan			All foreign laborers		
	Case	Population	Incidence^a^	Case	Population	Incidence
2004	16716	22689122	73.7	143	314034	45.5
2005	16472	22770383	72.5	168	327396	51.3
2006	15378	22876527	67.4	188	338755	55.5
2007	14480	22958360	63.2	190	357937	53.1
2008	14265	22037031	62	239	365060	65.5
2009	13336	23119772	57.8	193	351016	55.0
2010	13237	23162123	57.2	196	379653	51.6
2011	12634	23224912	54.5	257	425660	60.4
2012	12338	23315822	53	342	445579	76.8
2013	11528	23375517	49.4	340	489133	69.5
Average^b^	–	–	–	–	379422	–

^a^Presented with annual incidence with cases/ 10^5^

^b^The annual average numbers of the immigrant workers

**Table 2:** The incidence of tuberculosis among Taiwanese and the foreign laborers by different countries during 2004–2013, stratified by countries

	Indonesia laborers			Philippines laborers			Thailand laborers			Vietnam laborers		
	Case	Population	Incidence^a^	Case	Population	Incidence	Case	Population	Incidence	Case	Population	Incidence
2004	26	27281	95.3	41	91150	45.0	48	105281	45.6	28	90241	31.0
2005	19	49094	38.7	75	95703	78.4	51	98322	51.9	23	84185	27.3
2006	31	85223	36.4	72	90054	80.0	54	92894	58.1	31	70536	43.9
2007	56	115490	48.5	61	86423	70.6	41	86948	47.2	32	69043	46.3
2008	74	127764	57.9	68	80636	84.3	62	75584	82.0	35	81060	43.2
2009	77	139404	55.2	48	72077	66.6	34	61432	55.3	34	78093	43.5
2010	77	156332	49.3	56	77538	72.2	33	65742	50.2	30	80030	37.5
2011	111	175409	63.3	80	82841	96.6	37	71763	51.6	29	95643	30.3
2012	163	191127	85.3	75	86786	86.4	55	67611	81.3	49	100050	49.0
2013	152	213234	71.3	75	89024	84.2	55	61709	89.1	57	125162	45.5
Average^b^	–	128036	–	–	85223	–	–	78729	–	–	87404	–

^a^Presented with annual incidence with cases/ 10^5^

^b^The annual average numbers of the immigrant workers

Comparison of TB incidence among migrant workers and Taiwanese by different countries and genders are shown in Table 3. Comparing with Taiwanese by different countries, the TB risk was significantly higher in Philippines (HR: 1.22; 95%CI: 1.15–1.30, *P* < 0.001) but lower in Vietnamese (HR: 0.57; 95%CI: 0.47–68, *P* < 0.001). Comparing with Taiwanese by different genders, the TB risk was significantly lower in male migrant workers (HR: 0.76; 95% CI: 0.70–0.83, *P* < 0.001), but higher in female migrant workers (HR: 1.40; 95% CI: 1.35–1.46, *P* < 0.001).

**Table 3:** Comparison of TB incidence among foreign laborers and Taiwanese, stratified by gender, 2004 to 2013

	Tuberculosis cases	Average incidence^a^	Hazard ratio	*P* value
All				
Taiwan	140384	61.1	1.00	
All migrant workers	2255	58.4	0.97(0.93–1.01)	0.1846
Indonesia	786	60.1	1.00 (0.93–1.07)	0.9173
Philippines	606	76.4	1.22 (1.15–1.30)	<.0001
Thailand	514	61.2	0.98 (0.89–1.07)	0.6003
Vietnam	348	39.8	0.57 (0.47–0.68)	<.0001
Male				
Taiwan	95777	82.4	1.00	
All migrant workers	963	65	0.76 (0.70–0.83)	<.0001
Indonesia	180	98.8	1.18 (1.03–1.33)	0.0152
Philippines	199	69.6	0.83 (0.69–0.97)	0.0170
Thailand	407	61.8	0.71 (0.61–0.81)	<.0001
Vietnam	176	49.7	0.50 (0.35–0.64)	<.0001
Female				
Taiwan	42643	37.3	1.00	
All migrant workers	1292	55.9	1.40 (1.35–1.46)	<.0001
Indonesia	606	55.2	1.39 (1.31–1.47)	<.0001
Philippines	407	71.9	1.66 (1.56–1.75)	<.0001
Thailand	107	83.4	1.80 (1.62–1.99)	<.0001
Vietnam	172	33.1	0.88 (0.73–1.03)	0.1145

^a^Average Incidence was represented with annual cases per 10^5^ persons in 10 years average

Poisson regression model was applied to test the differences among each group

The TB risk was highest in 45–54 year-old group (49.6 per 10^5^) and lowest in ≤24 year-old group (12.4 per 10^5^) in Taiwanese while the TB risk was highest in 25–34 year-old group (65.9 per 10^5^) but lowest in 45–54 year-old group (30.8 per 10^5^) in migrant workers in Table 4. Applying Poisson regression models to test the differences among Taiwanese and migrant workers in each age group, we found that the TB risk in migrant workers was 5.30-fold in ≤24 year-old group comparing with Taiwanese (95%CI: 4.83–5.83, *P* < 0.001). Compared with Taiwanese, the TB risk of migrant workers in 25–34 year old and 35–44 year old groups are 2.63 fold (95%CI: 2.49–2.79, *P* < 0.001) and 1.40 fold (95%CI: 1.27–1.54, *P* < 0.001) respectively.

**Table 4:** Comparison of TB incidence among migrant workers and Taiwanese, stratified by age group, 2004 to 2013

	Tuberculosis Cases	Incidence^a^	Hazard ratio	*P* value
≤24 year-old				
Taiwanese	8862	12.4	1.00	
Migrant workers	457	65.9	5.30 (4.83–5.83)	<.0001
25–34 year-old				
Taiwanese	9631	25.2	1.00	
Migrant workers	1367	66.4	2.63 (2.49–2.79)	<.0001
35–44 year-old				
Taiwanese	12,203	32.7	1.00	
Migrant workers	427	45.7	1.40 (1.27–1.54)	<.0001
45–54 year-old				
Taiwanese	17,930	49.6	1.00	
Migrant workers	32	30.8	0.62 (0.44–0.88)	0.0071

^a^Average Incidence was represented with annual cases per 10^5^ persons in 10 years average

Poisson regression model was applied to test the differences among each group

## Discussion

To our knowledge, this is the first study to explore the TB incidence of the migrant workers in Taiwan. The nationwide, long-term follow-up, retrospective cohort study investigated the absolute risk and relative risk of TB in Taiwanese and the migrant workers. Gated by pre-screening program, the TB incidence in migrant workers was similar to Taiwanese but much lower than their native countries. Stratified by gender and age, the highest TB incidence group of the migrant workers were young female, especial in the ≤24 year-old group. There were two hypotheses to explain the differences of TB incidence between Taiwanese and migrant workers.

First hypothesis was that we assumed the high TB incidence after arrival of migrant workers was contributed by reactivation of latent TB infection (LTBI) [7, 11, 20]. In previous studies, the migrant workers, the refugees and the immigrants of US and Europe posed a high TB incidence after their arrival because the most important risk factor of reactivation of LTBI was that they were from countries of high tuberculosis burden [6, 21]. Studies suggest that active TB will develop in 5 to 10% of persons with LTBI during their lifetimes [22]. And, the highest risk period of reactivation among migrant workers was within first year and then during 2 to 4 years after their arrival [23, 24]. Also, the highest and lowest TB incidence of migrant workers in Taiwan were from Philippines and Vietnam while their native countries were at highest and lowest TB burden, respectively. In line with previous studies, our findings implied that there was a parallel risk of reactivation of LTBI based on the TB prevalence of the native countries [6, 7]. The current screening tool, chest radiography, was sensitive to detect pulmonary active TB but insufficient to catch LTBI [1, 25]. Our study pointed out that there was still high TB incidence among migrant workers after their arrival within 30 months. It implied that the current routine chest radiography in migrant workers in Taiwan was for screening pulmonary reactivation of LTBI but not adequate for detecting LTBI [12].

The second hypothesis of the high TB incidence after arrival of migrant workers was due to a new TB infection in Taiwan. Inconsistent with previous findings, which LTBI was more in male and elderly [21, 26], our study revealed that there was more TB cases in female and young group. There are three types of migrant workers in Taiwan: (1) those who work for companies and factories are classified as industrial and business workers, (2) those who work at house unit for cooking, clothing and child raising are classified as household workers, and (3) those who work in health care facilities or respiratory care centers are classified as health care workers [27]. Most of the household workers and health care workers were young and female which frequently contacted with the elderly and the persons with chronic illness which were groups with high proportion of tuberculosis infection. In line with previous systematic review and meta-analysis, there is a higher burden of LTBI among health care workers than general population in high burden countries [28]. Also, the matched cohort study in Taiwan revealed that the health care workers were in an increased risk of active TB compared with general population [29]. Besides, in previous studies in US and China, a proportion of migrant workers lived in crowded and poor surroundings [30, 31] which might increase the ongoing transmission of TB from cases of reactivation of LTBI to TB naive persons nearby among migrant workers. Furthermore, migrant workers experienced more stress during their daily work [32] and was less accessible to medical services which make them more vulnerable to infectious diseases. Because we did not performed polymerase chain reaction and bacterial cultures in each case, the active TB of young female migrant workers could be resulted from LTBI or a new infection after close contacts of persons with tuberculosis [21]. No matter the high TB incidence of the migrant workers were from LTBI or a new infection, they were all active in social community and easily spread the *Mycobacterium tuberculosis* to the public. Because the migrant workers are accounted for approximately 2% of total population in Taiwan, it is important to review the current screening program of migrant workers to improve TB control.

In Taiwan, once the migrant workers were diagnosed with new active TB, they have to receive 14-days treatments and repatriate back to their native countries [12]. Also, the migrant workers with active TB would be suspended from their work and quickly process the repatriation. Since cultured-based laboratory results require 6-weeks to final report, the migrant workers have relatively low culture data [27]. Nevertheless, chest X-ray is a valid and cost-saving screening tool for active and old healed TB, and is widely used in countries with migrant workers from high-burden areas [33]. The problem is that chest X-ray for active TB screening poses an unneglectable risk of onward reactivation of LTBI when migrant workers come from high TB risk countries [7]. Therefore, adding a screening tool of LTBI for high risk group in migrant workers is one of the applicable approaches in the future.

There are several limitation in our study. First of all, this was a retrospective cohort study and subjects’ information was obtained from the registration of local health administrations. Although we extracted several important confounding factors including origin countries, age and genders of migrant workers, the impact of unobserved and unmeasurable confounding factors such as histories of close contact with TB and underlying diseases of migrant workers cannot be ruled out. Second, the TB diagnosis in this study was based on either culture positive or radiological manifestation which might be not reflect the true tuberculosis infection. And, the proportion of positive TB smear or culture among reported active TB in migrant workers was only one third in Taiwan [27]. Besides, the tuberculosis cases of Taiwanese population were not detected by active screening which might cause an underestimation in the TB incidence in Taiwan. Furthermore, because all migrant workers were seronegative of HIV before their entry and were excluded from the study once diagnosis of HIV infection, the HRs of TB incidence might be higher than our estimation between the migrant workers and Taiwanese. In Taiwan, HIV is also a notifiable disease by the Law on the Control of Communicable Diseases in Taiwan. The annual reported new cases of HIV in Taiwan were 6.7–9.6 /per 10^5^. Due to the very low incidence of native HIV in Taiwan, we did not consider the problem of co- occurrence of TB and HIV [34].

However, this nationwide epidemiological investigation was consisted of whole population in Taiwan and all migrant workers from 2004 to 2013 which was representative and might provide a guide for further tuberculosis control in Taiwan.

## Conclusions

In conclusion, we demonstrated that the female and young migrant workers from high TB incidence countries were as a key reservoir of tuberculosis with consequently reactivation of LTBI and a probable risk of ongoing transmission in the first few years after their arrival. Our findings implied that this risky group should be prioritized to screen for LTBI as well as active TB in pre-arrival and post-arrival program. Further investigation and culture-based studies were warranted.

## Abbreviations

CIs: Confidence intervals
HRs: Hazard ratios
LTBI: Latent tuberculosis infection
NHI: National Health Insurance
TB: Tuberculosis
